# Continuous Processing Strategies for Amorphous Solid Dispersions of Itraconazole: Impact of Polymer Selection and Manufacturing Techniques

**DOI:** 10.3390/pharmaceutics17091090

**Published:** 2025-08-22

**Authors:** Madhuri M. Kshirsagar, Bandoo C. Chatale, Sathish Dyawanapelly, Lalitkumar K. Vora, Purnima D. Amin

**Affiliations:** 1 Department of Pharmaceutical Sciences and Technology, Institute of Chemical Technology, Nathalal Parekh Marg, Matunga, Mumbai 400019, MH, India; madhurikshirsagar.mk@gmail.com (M.M.K.); sa.dyawanapelly@ictmumbai.edu.in (S.D.); 2 Department of Pharmaceutical Chemistry, MET Institute of Pharmacy, Bandra Reclamation, Bandra, Mumbai 400050, MH, India; banduchatale89@gmail.com; 3 School of Pharmacy, Queen’s University Belfast, 97 Lisburn Road, Belfast BT9 7BL, UK

**Keywords:** amorphous solid dispersion, itraconazole, hot-melt extrusion, spray drying, solubility enhancement

## Abstract

**Background**: The limited aqueous solubility of BCS Class II drugs, exemplified by itraconazole (ITR), continues to hinder their bioavailability and therapeutic performance following oral administration. The present study investigated the development of amorphous solid dispersions (ASDs) of ITR via continuous manufacturing technologies, such as hot melt extrusion (HME) and spray drying (SD), to improve drug release. **Methods**: Polymer selection was guided by Hansen solubility parameter (HSP) analysis, film casting, and molecular modeling, leading to the identification of aminoalkyl methacrylate copolymer type A (Eudragit^®^ EPO), polyvinyl caprolactam–polyvinyl acetate–polyethylene glycol graft copolymer (Soluplus^®^), and hypromellose acetate succinate HG (AQOAT^®^ AS-HG) as suitable carriers. ASDs were prepared at drug-to-polymer ratios of 1:1, 1:2, and 2:1. Comprehensive characterization was performed using ATR-FTIR, NMR, DSC, PXRD, SEM, PLM, and contact angle analysis. **Results**: HME demonstrated higher process efficiency, solvent-free operation, and superior dissolution enhancement compared to SD. Optimized HME-based ASDs were formulated into tablets. The ITR–Eudragit^®^ EPO formulation achieved 95.88% drug release within 2 h (Weibull model, R^2^ > 0.99), while Soluplus^®^ and AQOAT^®^ AS-HG systems achieved complete release, best described by the Peppas–Sahlin model. Molecular modeling confirmed favorable drug–polymer interactions, correlating with the formation of stable complex and enhanced release performance. **Conclusions**: HME-based continuous manufacturing provides a scalable and robust strategy for improving the oral delivery of poorly water-soluble drugs. Integrating predictive modeling with experimental screening enables the rational design of ASD formulations with optimized dissolution behavior, offering potential for improved therapeutic outcomes in BCS Class II drug delivery.

## 1. Introduction

Oral drug delivery continues to be the most favored administration route in pharmaceutical formulations owing to its simplicity, patient adherence, cost efficiency, and adaptability in dosage form design [1,2]. However, the therapeutic efficacy of orally administered drugs is often limited by poor aqueous solubility, particularly for active pharmaceutical ingredients (APIs), which are classified as Biopharmaceutics Classification System (BCS) Class II. These compounds exhibit high permeability but low solubility, limiting their dissolution in gastrointestinal fluids and leading to abbreviated bioavailability [3].

ITZ is a broad-spectrum triazole antifungal drug widely used for treating superficial, subcutaneous, and systemic fungal infections [4,5,6]. As a BCS Class II compound with a high log P (3.7) and extremely low aqueous solubility (1–4 ng/mL), ITZ exhibits dissolution-limited absorption, resulting in poor and variable oral bioavailability [7,8,9,10]. Its highly crystalline nature further restricts its solubilization and dissolution, necessitating formulation strategies to overcome these limitations. Like other antifungal agents, ITZ functions by inhibiting fungal cytochrome P450 enzymes, disrupting sterol synthesis in the fungal cell membrane, and leading to cell death [11,12].

To overcome the solubility limitations of poorly water-soluble APIs such as ITZ, several formulation strategies have been explored, including salt formation [10,13], prodrugs [14], cocrystals [15,16], micelle systems [9] self-emulsifying drug delivery systems (SEDDSs) [17], particle size reduction [18], complexation [19], and amorphous solid dispersions [20]. Among these methods, the use of amorphous solid dispersions (ASDs) has emerged as a promising approach owing to their ability to disrupt the crystalline structure of APIs, thereby increasing their dissolution rate and bioavailability. ASDs are defined as homogeneous molecular dispersions of one or more APIs within an inert polymeric carrier, ensuring improved wettability and reduced particle size while inhibiting recrystallization [21,22].

ASDs confer kinetic and thermodynamic advantages by stabilizing the amorphous state of the API through polymer interactions, which hinder molecular mobility and nucleation [23]. However, the successful formulation of ASDs requires careful selection and optimization of formulation components to achieve a stable, homogenous dispersion [24,25]. Polymer selection plays a critical role, as it influences the miscibility of the drug–polymer system and prevents phase separation and drug crystallization [21,22]. Theoretical approaches such as the group contribution (GC) method, including Hansen solubility parameter calculations, are commonly employed to predict drug–polymer miscibility. Additionally, factors such as polymer viscosity, molecular weight, glass transition temperature (Tg), and chemical compatibility must be considered for ASD development [26,27].

Among the various techniques available for ASD preparation, solvent evaporation and melting processes are widely utilized [28]. Solvent evaporation, commonly implemented via spray drying (SD), involves dissolving the API and polymer in an organic solvent, followed by rapid solvent removal to form a solid dispersion. Transient solvent evaporation during the spray-drying process results in elevated viscosity and leads to the kinetic entrapment of the dispersed components inside the carrier matrix. The rapid evaporation of the solvent produces particles with a high surface area-to-volume ratio, resulting in enhanced dissolution [28,29,30].

Hot-melt extrusion (HME) working on the melting principle requires merely a unit step, wherein individual excipients can be directly introduced into the extruder barrel equipped with heating zones and rotating twin screws, facilitating the mixing of powders via heat and shear forces; consequently, pre-blending of the formulation components is rendered unnecessary [31,32,33]. Both SDs and HMEs have been extensively employed in the pharmaceutical industry for ASD manufacturing because of their continuous processability and scalability advantages, although HME is gaining prominence because of its solvent-free nature, enhanced content uniformity, and applicability to various dosage forms [34]. Although the intricate physicochemical characteristics of ASDs require multifaceted analytical tools to elucidate the correlations among formulations, process variables, and the in vitro performance characteristics of ASDs [23,35].

The current study aimed to investigate the physicochemical characteristics of ITZ-ASDs formulations with different polymeric carriers. The key objective of the present study was to design, develop, and compare both SD and HME continuous processing strategies for amorphous solid dispersions of ITZ and to further explore the impact of polymer selection and manufacturing techniques on the dissolution of ITZ.

## 2. Materials and Methods

### 2.1. Materials

Itraconazole (ITZ) was obtained from Virchow Healthcare Pvt. Ltd. (Hyderabad, India).

Aminoalkyl methacrylate copolymer type A (Eudragit^®^ EPO) was kindly gifted by Evonik Industries (India). Crospovidone (Kollidon^®^ CL), polyvinyl caprolactam–polyvinyl acetate–polyethylene glycol graft copolymer (Soluplus^®^), polyvinylpyrrolidone (Kollidon^®^ 30), propylene glycol (Kollisolv^®^ PG), polysorbate 80 (Kolliphor^®^ PS 80), and sorbitan monolaurate 20 (Kolliphor^®^ PS 20) were obtained from BASF India Ltd. (Mumbai, India). Hypromellose acetate succinate of HG grade (HPMC AS, AQOAT^®^ AS-HG) was provided as a gift sample by Shin-Etsu Chemical Co., Ltd. (Tokyo, Japan). Triethyl citrate was procured from S.D. Fine-Chem Ltd. (Mumbai, India). Directly compressible mannitol (PEARLITOL^®^ 200 SD) was supplied by Roquette Pharma (Lestrem, France). Magnesium stearate (EMPROVE^®^ Essential) was obtained from Merck (Mumbai, India). Microcrystalline cellulose (Avicel^®^ PH-102) was received as a gift sample from IFF Pharma Solutions (Wilmington, NC, USA). Dichloromethane, methanol, and deuterated dimethyl sulfoxide (DMSO-d6), all of analytical grade, were utilized.

### 2.2. Methods

#### 2.2.1. Selection of Formulation Components for the ASD

Generally, an ASD is a thermodynamically unstable system, so selecting components within the same system becomes crucial [36]. Consequently, an initial qualitative and quantitative selection of formulation components for ASD was conducted, emphasizing physicochemical interactions and system stability.

##### Hansen Solubility Parameter

Miscibility interactions between the drug and ASD polymers were evaluated via the Hansen solubility method, as described by Hoftyzer and Van Krevelen. Hansen solubility parameter (HSP) values are quantitative assessments of dispersion forces (δD), polar forces (δP), and hydrogen bonding forces (δH) in the formulation components to ascertain miscibility behavior. Substances exhibiting comparable HSP values are usually more prone to mutual solubility, whereas those with significant disparities in HSP values are less likely to be miscible.

The HSP value is calculated via the following equation:

δ_T_^2^ = δ_D_^2^ + δ_P_^2^ + δ_H_^2^ (1)

where δ_T_ is the HSP value; δ_D_ is the dispersion component, which represents nonpolar or van der Waals forces; δ_P_ is the polar component, which represents dipole-dipole or polar forces; and δ_H_ is the hydrogen bonding component, which represents hydrogen bonding forces. These soluble components can be determined on the basis of the structural groups present in the molecule, as reported in the literature [37,38,39,40].

##### Film Casting Method

This technique determines the ability of polymers to solubilize drugs and their recrystallization inhibition behavior. The drug was taken with various polymers, including Soluplus^®^, Eudragit^®^ EPO, Kollidon VA 64, HPMC AS LG, HPMC AS MG, and AQOAT^®^ AS-HG at the ratios 1:1, 1:2, and 2:1 and dissolved in a common volatile solvent. After complete solubilization, the solution was poured into a Petri plate, which was dried at room temperature. As the solvent evaporated, a thin layer on the Petri plate was obtained from a mixed composition of the drug and polymer. The formation of a transparent film indicated the homogenization of the API with the polymer, whereas opaque visualization indicated phase-separation behavior. Large amounts of drugs can be dissolved in polymers with increased solubilization capabilities. The film was again dispersed in dichloromethane, dried, and then inspected to identify the inhibition effectiveness of the polymer towards the recrystallization of API [41,42].

##### Computational Studies for the Optimization of ASD Components

The molecular modeling study was performed by using Schrodinger Material Science Suite 2024-3. The chemical structures of Itraconazole, AQOAT^®^ AS-HG, Eudragit^®^ EPO, and Soluplus^®^ were drawn via a 2D sketch of Schrodinger Maestro 2024-3. The structures were optimized for Jaguar studies via the Ligprep module of Schrodinger. The structures were converted into 3D and energy-optimized via OPLS 2004. The least energy-based conformations were generated and used for further studies. The solvation energies for itraconazole, AQOAT^®^ AS-HG, Eudragit^®^ EPO, and Soluplus^®^ were predicted via the Jaguar module of the Material Science Suite of Schrodinger Maestro 2024-3. The binding energy (Ebinding) for each combination (1:1, 1:2, and 2:1) was computed using the formula E_binding_ = E_complex_ − ∑ (E_ingredients_), where E_complex_ is the energy of the drug–polymer system, and *∑* (*E_ingredients_*) is the sum of individual components in stoichiometric proportions [43,44].

#### 2.2.2. Development of Amorphous Solid Dispersions Through Spray Drying

For this process, the first molecularly dispersed feed solution was prepared by dissolving the drug and ASD polymer into a volatile solvent. The physical mixtures of ITR with Eudragit^®^ EPO, Soluplus^®^, and HPMC AS were taken at different ratios of 1:1, 1:2, and 2:1. The prepared mixtures were then dissolved in a mixture of dichloromethane and methanol (50:50), and the solvents used for spray drying were selected in accordance with the methods of the film casting studies [45,46,47]. The mixtures were then spray dried in a Unispray Spray Dryer Lab model (S B Panchal & Company, Mumbai, India) to obtain free-flowing powders. The process parameters, such as the atomization pressure (kg/cm^3^), aspiration rate (Nm^3^/h), and inlet temperature, and the material properties, such as the solid concentration (% *w*/*v*) and drug/polymer ratio, were optimized to achieve a high yield, high solubility, and good drug release.

#### 2.2.3. Development of Amorphous Solid Dispersions Through HME

The drug was mixed gently yet homogenously with the polymers and plasticizer in specific geometric ratios in a mortar with a pestle to prepare physical mixtures (PMs) of 10 g each. ITR was mixed with Eudragit^®^ EPO, Soluplus^®^, and AQOAT^®^ AS-HG at ratios of 1:1, 1:2, and 2:1. A total of 5% plasticizer, including trimethyl citrate, propylene glycol, polysorbate 80, and sorbitan monolaurate 20, was subsequently added to the physical mixture system for process feasibility. The extrusion temperatures were selected on the basis of the melting temperature, glass transition temperature, and degradation temperature of the API and polymer. The powder blends were fed in small amounts into a fully intermeshing mini twin-screw extruder (Thermo Scientific, HAAKE MiniCTW, Karlsruhe, Germany) equipped with conical, counter-rotating, and stainless steel-coated screws. Processing parameters such as the screw speed, temperature, torque, and residence time were optimized concerning process feasibility, amorphous state conversion, drug content, and drug release. All the physical mixtures were processed at various extrusion temperatures of 150, 160, and 170 °C and screw speeds of 20, 40, and 60 rpm.

#### 2.2.4. Preparation of Tablets with Optimized ASD Formulations

The refined amorphous solid dispersions (ASDs) of itraconazole (equivalent to 100 mg) were subsequently compacted into tablets for in vitro dissolution and release kinetics studies. Each formulation was blended with appropriate tablet excipients, including Kollidon^®^ CL (superdisintegrant), Kollidon^®^ 30 (binder), PEARLITOL^®^ 200 SD mannitol (diluent), Avicel^®^ PH 102 (filler), and magnesium stearate (lubricant). The blend was uniformly mixed using a mortar and pestle to ensure homogeneity. The final mixture was compressed into tablets weighing 500 mg using a single-punch Eliza-press machine (EP-200 L, Mumbai, India) fitted with 9 mm flat-faced punches.

To elucidate the drug release mechanisms from the ASD-loaded tablet formulations, the in vitro dissolution profiles of the optimized batches were subjected to comprehensive kinetic modeling. Determining drug release kinetics provides insight into the dominant release mechanism, such as diffusion, erosion, or anomalous transport. In addition to classical models such as zero-order, first-order, Higuchi, and Korsmeyer–Peppas, extended models including the Hixson–Crowell, Hopfenberg, Weibull, and Peppas–Sahlin models were applied to evaluate release kinetics. Modeling was performed using DDSolver 1.0 software. Kinetic parameters such as the release rate constant (k), correlation coefficient (R^2^), shape factor (β), and release exponent (n) were determined and compared across models to identify the best-fitting kinetic model for each formulation.

#### 2.2.5. Characterization of Itraconazole Solid Dispersions

##### Attenuated Total Reflection Fourier Transform Infrared Spectroscopy (ATR-FTIR)

The samples were examined via attenuated total reflection Fourier transform infrared (ATR-FTIR) spectroscopy FTIR/4600 spectrometer (Jasco Co., Tokyo, Japan) with a ZnSe prism and a TGS detector operating in the 4000–400 cm^−1^ range was used to collect the spectra. The raw spectra were subjected to ATR correction using Jasco Spectra Manager version 2.

##### DSC Analysis

Thermal analysis was conducted via DSC (DSC-60, Shimadzu, Kyoto, Japan) to ascertain the physical state of the ITR inside the polymer matrix and assess any potential crystallization behavior. The API and optimized ASDs were sealed in aluminum pans and subjected to DSC under a 100 mL/min nitrogen flow rate. The thermograms were obtained by subjecting the samples to a heating rate of 10 °C/min, ranging from 25 to 250 °C.

##### PXRD Analysis

The crystallinity of the ITR and optimized ASDs was assessed via a Brucker D2 Phaser 2nd Generation X-ray diffractometer (Karlsruhe, Germany) operating at a voltage of 30 kV, a current of 10 mA, a scanning rate of 15.00°/min, and a 2θ range of 5.002°–60.990°, with an angular accuracy of ±0.02° 2θ, and it was further analyzed via the DIFFRAC. SUITE MEASUREMENT CENTER 21 CRF Part 11 V7.5.

##### ^1^H Nuclear Magnetic Resonance (NMR) Analysis

The ^1^H NMR spectra of the API, polymer, and solid were obtained in deuterated DMSO, i.e., DMSO-d^6^, with tetramethylsilane as an internal standard, via an Agilent 500 MHz spectrometer (Agilent Technologies, Hambuecker Landstrasse, Waghäusel-Wiesental, Germany) at 25 °C. The coupling constants (J) are in hertz (Hz) units. The measured spectra were analyzed via MestReNova software (version: 6.0.2--5475). The accuracy of the ^1^H NMR was < 0.01 ppm, and the sensitivity of the instrument was (S/N) 374:1.

##### Polarizing Light Microscopy

The morphology and microstructure of the SD and ITR samples were analyzed at 10× magnification via a polarizing microscope (LEXT^®^ OLS 5000) (Mumbai, India). This technique allows the visualization of crystallite formation through birefringence, thus identifying drug crystallization from polymeric matrices.

##### Scanning Electron Microscopy (SEM) Analysis

A scanning electron microscope (FEI Quanta 200 FEG) equipped with a 15 kV accelerating voltage was used to compare the surface morphologies of the pure ITZ, optimized solid dispersion strands, and milled solid dispersion. To secure the samples to the SEM stubs, double-sided adhesive tape was used. The obtained micrographs were examined at a magnification ratio of ×1000.

##### Contact Angle

The contact angle is an often-used approach for assessing the wettability of a surface or substance, which is related to the deposition of liquid on a solid substrate and its capacity for forming boundary interfaces with it [48,49,50]. The contact angle measurements of the API and optimized ITR ASDs were conducted via the sessile drop methodology with the half-angle fitting method, which employs an APEX contact angle meter (ACAM NSC Series) with an angular accuracy of ±0.05°. The powder samples were positioned on a level surface affixed with double-sided adhesive tape. A drop of distilled water was applied to the sample via a syringe positioned 0.5 cm above it. Contact angle measurements were conducted in triplicate within 10 ± 3 s.

##### In Vitro Drug Release Study

In vitro drug release studies were conducted to evaluate the dissolution behavior of plain itraconazole (ITR) and its amorphous solid dispersions (ASDs) prepared with Eudragit^®^ EPO, Soluplus, and HPMC-AS. Experiments were performed using the USP type-II Dissolution Apparatus, Mumbai, India (paddle method, LABINDIA DS 8000 eight-station dissolution system). ASDs containing Eudragit^®^ EPO and Soluplus were tested in 0.1 N HCl (pH 1.2) [51] to simulate gastric conditions, whereas HPMC-AS ASDs were evaluated in distilled water containing 0.5% *w*/*v* sodium lauryl sulfate (SLS) to mimic intestinal-like solubilization conditions (pH~6.8) [52]. The paddle rotation speed was set at 100 rpm for pH 1.2 and 75 rpm for pH 6.8, and the temperature of the dissolution medium was maintained at 37 ± 0.5 °C. Dissolution studies were carried out using pure ITR powder (100 mg) and ASD powders equivalent to 100 mg of ITR, sieved to a uniform particle size (passed through #40 mesh and retained on #60 mesh) to ensure consistent surface area exposure. Additionally, optimized ASD tablet formulations were also evaluated under the same conditions. At predetermined time intervals (5, 15, 30, 45, 60, 90, and 120 min), 5 mL aliquots were withdrawn, filtered, and replaced with fresh medium to maintain sink conditions. The samples were appropriately diluted and analyzed using a Shimadzu UV-2600 spectrophotometer at 256 nm for 0.1 N HCl and 272 nm for 0.5% SLS solution. The UV spectrophotometric method was validated following ICH Q2(R1) guidelines.

## 3. Results and Discussion

### 3.1. Selection of Formulation Components for the ASD

#### 3.1.1. Hansen Solubility Parameter

The total solubility parameter difference values (Δδ) between the solute (ITR) and solvents (ASD polymers), expressed in megapascals (MPa), were used to assess the miscibility between formulation components. The values between the ITR and the polymers are displayed in Table 1. All the total solubility parameter difference values (Δδ) between the ITR and the polymers were less than 7 MPa½, indicating the presence of miscible systems. The highest Δδ value among the analyzed polymers was noted between ITR and Eudragit^®^ EPO, followed by Soluplus^®^, HPMC AS LG, HPMC AS MG, Kollidon VA 64, and AQOAT^®^ AS-HG. The Δδ of the AQOAT^®^ AS-HG with an ITR was substantially lower than that of the other polymers, indicating better miscibility of the polymer [53,54]. However, this approach provides only qualitative predictions of solubility, rendering the resulting data inadequate for forecasting composition-based miscibility. Consequently, to investigate the API loading ratio and solubilization capability of the polymers, the solvent casting approach was utilized.

#### 3.1.2. Film Casting Method

Solvent casting indicated that all the polymers with an API/polymer ratio of 1:2 were capable of solubilizing drugs at both the primary and secondary stages (Table 2). However, at a 1:1 ratio, only Soluplus^®^ and AQOAT^®^ AS-HG showed complete miscibility of API at both drying stages; Kollidon VA 64 and AQOAT^®^ of AS-HG grade produced a transparent film at the primary stage but became slightly turbid at the secondary stage, which indicated recrystallization of the API. Among the different grades of HPMC AS, AQOAT^®^ AS-HG possesses a relatively high degree of hydrophobic substitution (increased acetyl content), offering favorable binding sites for lipophilic drugs. This property enhances its effectiveness in preventing drug crystallization, making it a superior stabilizing agent for amorphous formulations [55].

Considering the higher drug loading in the drug/polymer ratio of 2:1, all the polymers were found to be incapable of completely solubilizing API and inhibiting the crystallization phenomenon of API. Thus, the results obtained from the film casting method were in agreement with Hansen’s solubility parameters. On the basis of these results, the polymers Soluplus^®^, Eudragit^®^ EPO, and AQOAT^®^ AS-HG were selected for further studies.

#### 3.1.3. Computational Studies for the Optimization of ASD Components

ITR was modeled in combination with selected polymers—Soluplus^®^, Eudragit^®^ EPO, and AQOAT^®^ AS-HG—at drug-to-polymer ratios of 1:1, 1:2, and 2:1. Energy calculations were conducted for both gas-phase and solution-phase states.

Gas-phase energy refers to the energy of a solute in its isolated molecular form, indicating intrinsic molecular stability. Lower gas-phase energies imply greater intrinsic stability, which may enhance the potential for dissolution. Conversely, solution-phase energy incorporates solute–solvent interactions. However, total solution-phase energy values for higher drug or polymer ratios are generally not normalized per mole and reflect cumulative system size [56,57]. Thus, binding energy (E_binding_) was also calculated to quantify interaction strength: Ebinding = E_complex_ − Σ(E_ingredients_), where E_complex_ is the energy of the drug–polymer complex system, and Σ(E_ingredients_) is the sum of energies of the individual drug and polymer components. Table 3 summarizes the gas-phase energy, solution-phase energy, and computed binding energy for ITR, polymers, and their respective combinations:

##### Analysis of Itraconazole and Eudragit^®^ EPO Combinations

The combination of itraconazole (ITR) with Eudragit^®^ EPO demonstrated favorable thermodynamic behavior across all evaluated ratios. At the 1:1 ratio, the gas-phase and solution-phase energies were −4289.00 kcal/mol and −4289.10 kcal/mol, respectively, with a moderately negative binding energy of −292.82 kcal/mol, indicating a stable drug–polymer interaction. Increasing the polymer content to a 1:2 ratio further reduced both gas- and solution-phase energies (−5753.76 kcal/mol), while the binding energy became highly negative (−777.70 kcal/mol), suggesting stronger molecular interactions and a favorable environment for drug solubilization. At the 2:1 ratio, although total system energies were lowest (gas: −6482.18 kcal/mol; solution: −6482.46 kcal/mol), the binding energy turned strongly positive (+530.32 kcal/mol), indicating that the higher drug load may exceed the polymer’s capacity to maintain a solubilized and compatible state.

##### Analysis of Itraconazole and Soluplus^®^ Combinations

For Soluplus^®^, gas-phase and solution-phase energies decreased progressively with increasing drug/polymer ratios. The 1:1 combination resulted in energies of −4263.75 kcal/mol (gas) and −4263.88 kcal/mol (solution), with a binding energy of +204.59 kcal/mol, reflecting weak interaction. At 1:2 and 2:1 ratios, the gas-phase energies were −5919.82 kcal/mol and −7483.81 kcal/mol, and solution-phase energies were −5919.90 kcal/mol and −7483.93 kcal/mol, with correspondingly low positive binding energies of +0.54 kcal/mol and +1.04 kcal/mol. While specific binding remains limited, the consistent reduction in total system energy at higher ratios suggests improved compatibility, supporting Soluplus^®^’s utility as a dispersing carrier in amorphous solid dispersions.

##### Analysis of the Effects of the Combination of Itraconazole and AQOAT^®^ AS-HG

The ITR combinations with AQOAT^®^ AS-HG (HPMC AS) exhibited a similar thermodynamic profile. At the 1:1 ratio, gas- and solution-phase energies were −6383.34 kcal/mol and −6383.47 kcal/mol, respectively, with a slightly positive binding energy of +74.47 kcal/mol, likely due to limited polymer presence. The 1:2 ratio showed the most favorable interaction, with highly negative energies in both phases (gas: −9981.21 kcal/mol; solution: −9981.43 kcal/mol) and a negative binding energy of −82.05 kcal/mol, reflecting effective drug–polymer association and improved conditions for solubilization. At the 2:1 ratio, the system also remained thermodynamically favorable (binding energy: −76.19 kcal/mol), indicating that higher drug loading can be accommodated when an adequate polymer is available to sustain dispersion.

### 3.2. Development of Amorphous Solid Dispersions Through Spray Drying

The spray-drying parameters for preparing ASD were optimized by systematically adjusting one parameter at a time. Spray-dried solutions were prepared with 8–10% solids content (*w*/*w*) with varying ratios of ITR and ASD polymers in a 1:1 (*w*/*w*) solution of dichloromethane and methanol. The solutions were then pumped into the atomizer at a rate of 20 mL/min. The inlet and outlet temperatures for Soluplus^®^ ITR SD, Eudragit^®^ EPO SD, and AQOAT^®^ AS-HG SD were 60 ± 5 °C and 32 ± 5 °C, 65 ± 6 °C, and 35 ± 5 °C and 80 ± 4 °C and 55 ± 5 °C, respectively. Aspirator control and atomization pressures were optimized to 20 Nm^3^/h and 1 bar, respectively.

### 3.3. Development of Amorphous Solid Dispersions Through HME

Initially, the samples were extruded at three different temperatures (150 ± 4, 160 ± 4, and 170 ± 4 °C) while maintaining the same screw speed (20 rpm).

For the ITR Eudragit^®^ EPO ASD at 150 °C for all API/polymer ratios, opaque extrudes were obtained. For ITR EPO ratios of 1:1 and 1:2, clear extrusion was obtained at 160 °C, although the former ratio turned opaque after a few min, indicating recrystallization behavior and possibly minimal miscibility between the API and the polymer. Extrusion of 1:1 and 2:1 ratios was possible at 170 °C with transparent extruders. However, the 2:1 ratio batch immediately underwent recrystallization, showing the inadequate capacity of the polymer to solubilize API even at high temperatures. Similarly, for the ITR/Soluplus^®^ ASDs at 150 °C for all API/polymer ratios, opaque extrudes were obtained. At 160 °C, clear transparent extrudes were obtained for all the API/polymer ratios without turning opaque. This finding highlights the excellent solubilization capacity and processing feasibility of Soluplus^®^. For the ITR:AQOAT^®^ AS-HG, ASDs at 150 °C and 160 °C were unsuitable for producing clear transparent extrudes without recrystallization. At 170 °C, clear extrusion of ITR:AQOAT^®^ AS-HG was obtained at all ratios, although a 2:1 ratio batch of ITR:AQOAT^®^ AS-HG underwent recrystallization after a few hours.

**Figure 1:**
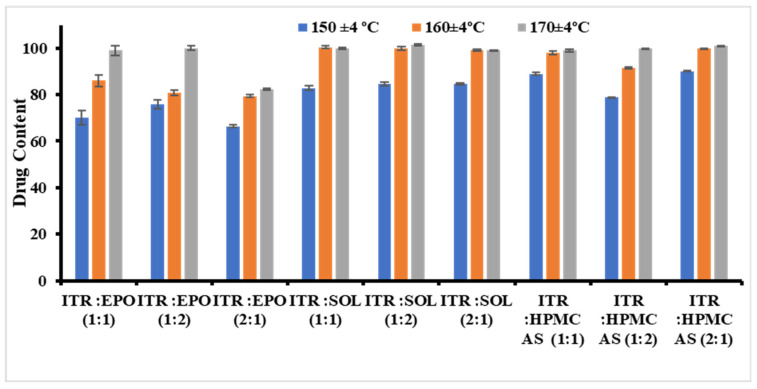
Effect of temperature on the drug content (mean values, ±SDs) in the development of ASD (n = 3).

The effects of changes in the above parameters, such as homogeneity, content uniformity, and drug content, on the formulation properties were thoroughly studied. Compared with 150 °C, temperatures between 160 °C and 170 °C during the extrusion process yielded better drug contents and enhanced physical and chemical uniformity. This improvement may be attributed to the increased fluidization of the polymer chains at elevated temperatures, which facilitates better mixing and melting, thereby enhancing the homogeneity and drug distribution [58,59]. For the ITR EPO and ITR AQOAT^®^ AS-HG solid dispersions, the optimal drug content was observed at 170 °C, and for the ITR SOL solid dispersions, the optimal drug content was observed at 160 °C (Figure 1). Thus, temperature was identified to play a crucial role in the development of ITR ASDs. After the processing temperatures (170 °C for Eudragit^®^ EPO ASDs, 160 °C for Soluplus^®^ ASDs and 170 °C for AQOAT^®^ AS-HG ASDs) were fixed, different screw speeds were evaluated. No major effect of the screw speed on the product performance characteristics was observed.

However, relevant observations were made with respect to drug loading and torque. For all the polymers, it was observed that with higher drug loading, there was a reduction in the torque value; possibly because of the plasticizing effect of itraconazole [60] (see Figure S1 in Supplementary Materials). Additionally, a considerable inverse relationship between temperature and torque was observed (see Figure S2 in Supplementary Materials).

### 3.4. Characterization of Itraconazole Solid Dispersions

#### 3.4.1. Attenuated Total Reflection Fourier Transform Infrared Spectroscopy (ATR-FTIR)

**Figure 2:**
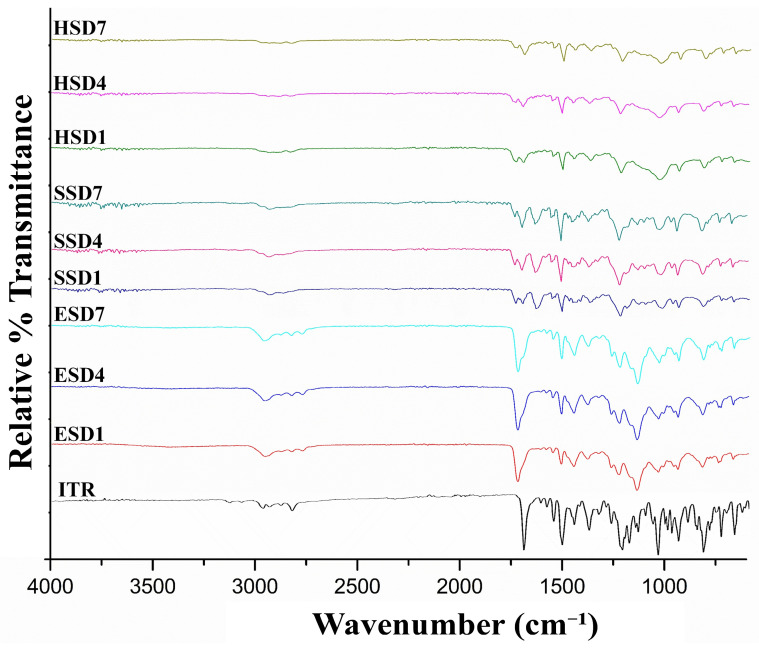
ATR-FTIR spectra of the ITR and ASD.

FTIR spectroscopy was employed to investigate possible drug–polymer interactions in the amorphous solid dispersions (Figure 2). Pure itraconazole exhibited characteristic vibrational bands at 1708 cm^−1^ (C=O stretching) [61], 1514 cm^−1^ (C=N stretching) [62], and 1140–1250 cm^−1^ (C–O–C stretching) [63,64,65]. In the ASD formulations, these bands showed noticeable shifts and/or broadening, particularly in the carbonyl and amine regions, indicating the formation of hydrogen bonding or other intermolecular interactions between the drug and polymer matrix, crucial for the formation of amorphous products.

#### 3.4.2. DSC Analysis

**Figure 3:**
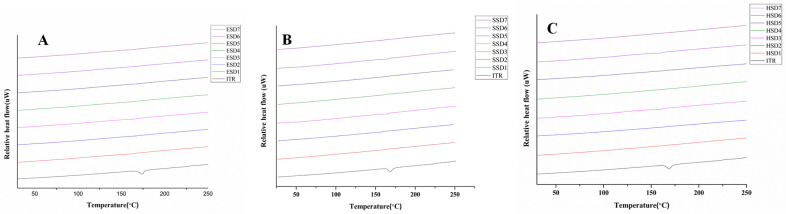
DSC spectra of optimized ASDs (**A**–**C**); ITR EPO ASDs (**A**), ITR SOL ASDs (**B**), and ITR AQOAT^®^ AS-HG ASDs (**C**).

DSC analysis was performed to investigate any changes in the physical state of the drug and the optimized ASDs used in the study. The results are shown in Figure 3A–C. ITR showed a sharp endothermic peak at approximately 169 °C, which corresponded to its melting point [62,66,67]. Notably, the endothermic peak of the ITR completely disappeared in the ITR ASDs, indicating the amorphous conversion of API and the dissemination of API in the polymer matrix during spray drying and the hot-melt extrusion process [68]. PXRD was used to further validate the above results.

#### 3.4.3. PXRD Analysis

**Figure 4:**
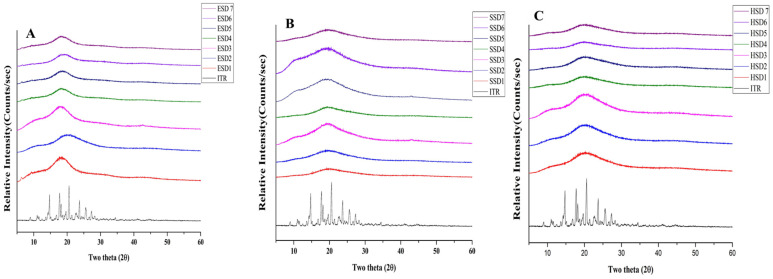
PXRD spectra of optimized ASDs: ITR EPO ASDs (**A**), ITR SOL ASDs (**B**), and ITR HPMC AS ASDs (**C**).

The presence of well-defined PXRD peaks in the ITR sample suggested its crystalline structure. PXRD analyses of the optimum ITR ASDs revealed the absence of distinctive peaks associated with the ITR at 2θ values of 14.216, 14.743, 17.766, 18.210, 19,679, 20.585, 22.712, 23.704, 25.495, and 27.352 (Figure 4A–C). In contrast, the ASD formulations showed a complete absence of these peaks and displayed a broad halo between 15° and 25° 2θ, indicating successful amorphization of itraconazole, consistent with previous reports where similar PXRD patterns were observed for amorphous itraconazole systems [16,47,62,69]. The absence of any melting endotherm in DSC thermograms further supports the complete amorphous conversion.

#### 3.4.4. ^1^H Nuclear Magnetic Resonance (NMR) Analysis

**Figure 5:**
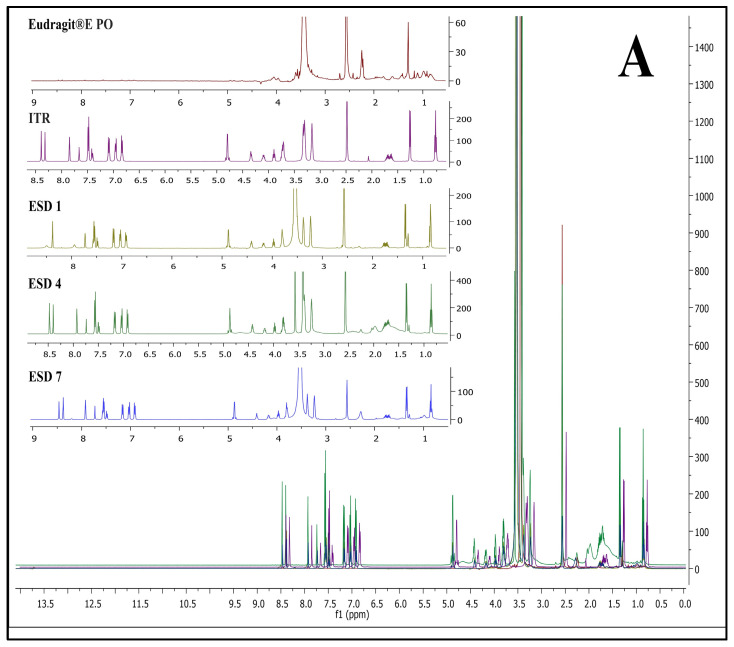

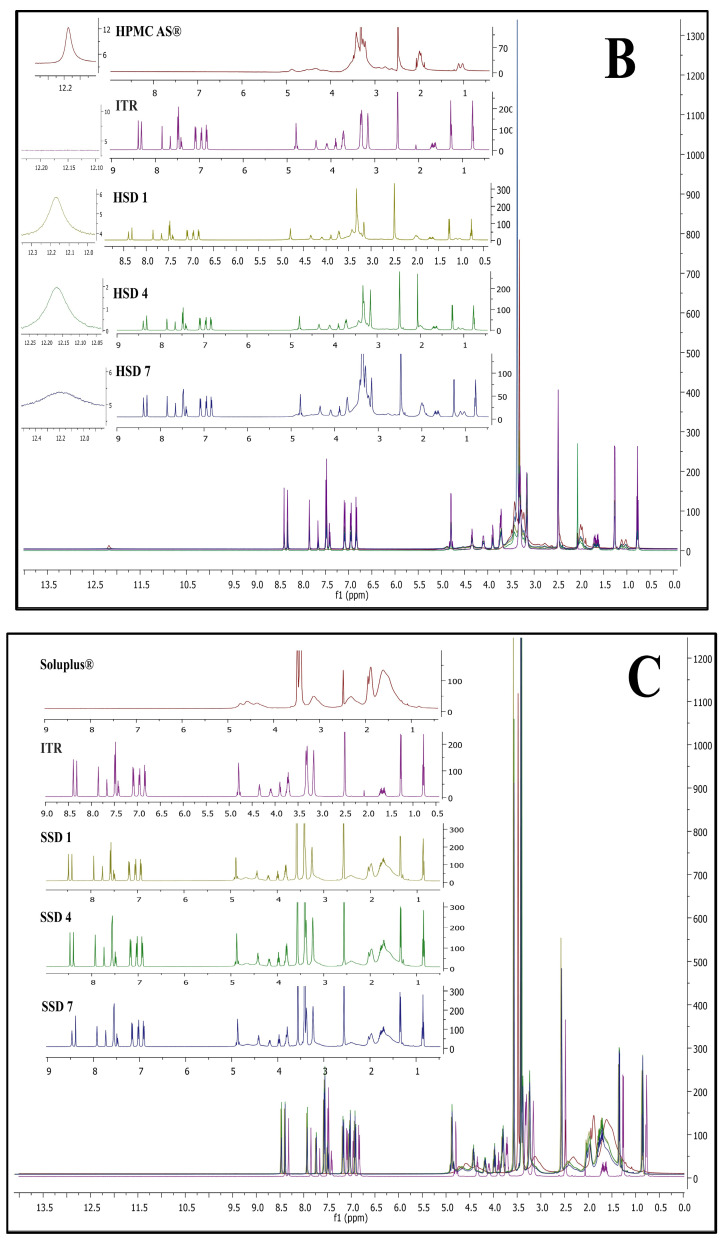
NMR spectra of ITR EPO ASDs (**A**), ITR SOL ASDs (**B**), and ITR HPMC HG ASDs (**C**).

One of the straightforward experimental approaches currently used to characterize novel ASDs includes ^1^H NMR. Chemical shielding interactions have been demonstrated to be very sensitive to drug–polymer interactions and subtle changes in the local electronic environment, indicating the occurrence of API-polymer interactions in ASDs [50,51,52]. Figure 5 represents NMR spectra of ITR EPO ASDs (A), ITR SOL ASDs (B), and ITR HPMC HG ASDs (C).

In ITR EPO ASDs, the ^1^H NMR signals were observed to shift downfield compared with the signals present in the polymer and ITR individually. Additionally, one crucial finding was observed: a peak near 2.25 ppm from the polymer Eudragit EPO was nearly suppressed in ITR EPO-loaded ASDs. These observations may be attributed to the potential hydrogen bonding interaction between the ITR and the latter [70,71,72]. The intensities of the proton signals in ESD7 were diminished due to the inclusion of the plasticizer propylene glycol, possibly improving the binding interactions relative to those in ESD4. Similar to ITR EPO ASDs, a downfield shift in the chemical signals was observed for the ITR Soluplus^®^-loaded ASDs in comparison with the ITR and the Soluplus^®^ polymer.

The chemical shift for signals was minimally observed in ITR HPMC-AS-loaded ASDs, likely due to steric hindrance contributed by the polymer’s acetyl and succinyl groups, an effect potentially intensified by the presence of heterocyclic and carboxylic groups in ITR [73]. Additionally, the acid-base interactions among acidic polymers and basic API moieties also strongly contribute to API stabilization in ASDs, and these interactions are considerably stronger than H-bonds [73,74,75,76]. Conversely, hydrogen bonding is evident in the ITR EPO ASDs and ITR Soluplus^®^ ASDs, as indicated by the chemical shift resulting from minor alterations in the surrounding environment of the protons engaged in these interactions. In the ITR HPMC AS HG, a prominent singlet signal at approximately 12.2 ppm was observed for the acidic proton (carboxylic acid group) of the polymer (height was observed to be 12 on the intensity scale), which was found to be strongly suppressed in the case of the ASDs. In the case of HSD 1 and HSD 4 (spray-dried and HME batches, respectively), both batches showed substantial peak broadening as well as suppression of peak height intensity. However, the peak intensity of the carboxylic acid group of HPMC-AS was greater for HSD 4 than for HSD 1 (intensity of 2 for HSD 4 versus 6 for HSD 1). This suggests that, compared with spray drying, the HME process facilitated stronger binding interactions within the API-polymer matrix. In HSD 7, increased suppression was observed, which is attributable to improved molecular interactions due to the presence of the plasticizer propylene glycol.

#### 3.4.5. Polarizing Light Microscopy

**Figure 6:**
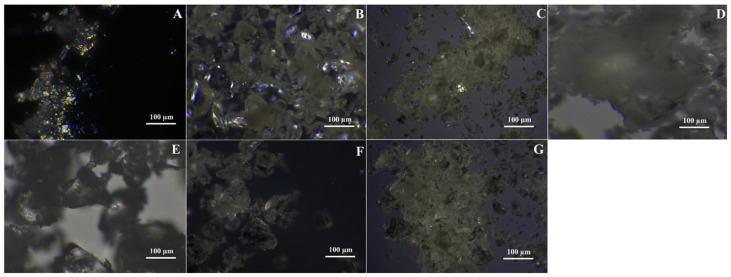
Polarizing light microscopy (PLM) images of the ITR and optimized ASDs: (**A**) = ITR, (**B**) = ESD1, (**C**) = SSD1, (**D**) = HSD1, (**E**) = ESD4, (**F**) = SSD4, and (**G**) = HSD4.

Micrographs under polarized microscopy comparing the ASDs with the corresponding API at different total drug loading levels are presented in Figure 6A–G. The crystalline ITR clearly shows typical crystalline birefringence with a flake-like particle morphology. Minimal birefringence was observed in the spray-dried batches of ITR EPO and ITR Soluplus^®^, suggesting the potential initiation of crystallite formation [77]. No such occurrence was found for the spray-dried ITR HPMC HG solid dispersion, suggesting the recrystallization inhibition potential of the solid dispersion carrier. Similar behavior was noted with ITR ASDs produced with HME, where irregularly shaped, glass-like particles devoid of birefringence were detected, showing the superiority of the HME technique for molecular-level mixing, resulting in the development of ASDs [78,79] (Figure 6).

#### 3.4.6. Scanning Electron Microscopy (SEM)

**Figure 7:**
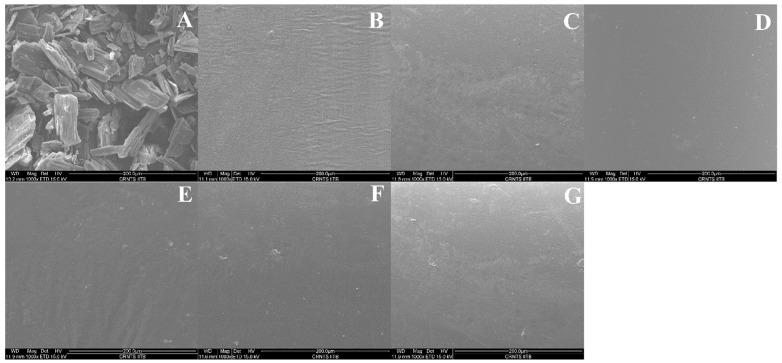
Scanning electron micrographs (SEM) of the ITR and optimized ASDs: (**A**) = ITR, (**B**) = ESD1, (**C**) = SSD1, (**D**) = HSD1, (**E**) = ESD4, (**F**) = SSD4, and (**G**) = HSD4.

Scanning electron micrographs of the solid dispersions prepared with Soluplus^®^, Eudragit^®^ E PO, and AQOAT^®^ AS-HG are shown in Figure 7A–G at a magnification ratio of ×1000. Pure itraconazole was irregular in its crystalline shape, whereas the solid dispersion strands were found to have a plane, dense, even, compact surface that was devoid of visible crystals, indicating uniform dispersion of API into the polymer.

#### 3.4.7. Contact Angle

**Figure 8:**
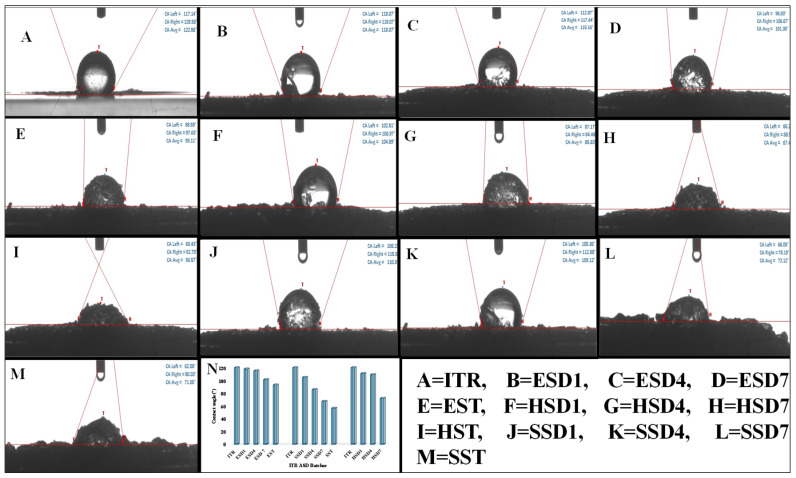
Contact angle analysis for ITR (**A**), ITR EPO ASDs (**B**–**D**), ITR EPO ASD Tablets (**E**), ITR HPMC AS ASDs (**F**–**H**), ITR HPMC AS ASD Tablets (**I**), ITR Soluplus^®^ ASDs (**J**–**L**), ITR Soluplus^®^ ASD Tablets (**M**), and comparison of ASD batches (**N**).

As shown in Figure 8, contact angles for ITZ were observed to be 122°, which indicated the extremely hydrophobic nature of the drug. Among all the batches, spray-dried batches (ESD1, SSD1, and HSD1) presented contact angles comparable to those of HME batches (ESD4, SSD4, and HSD4), except for ITR Soluplus^®^ ASD batches, which presented minimal contact angles, which were further reduced by the addition of plasticizers, increasing the hydrophilicity of the system (SDS7 and HSD7 in batches). The wettability investigation of the tablet blend revealed a greater reduction in the contact angle, which was attributable to the inclusion of additional hydrophilic matrix-forming excipients in the tablet formulation.

As shown in Figure 8, the contact angle for ITZ was 122°, which indicated the extremely hydrophobic nature of the drug. Among all the batches, spray-dried batches (ESD1, SSD1, and HSD1) presented contact angles comparable to those of HME batches (ESD4, SSD4, and HSD4), except for ITR Soluplus^®^ ASD batches, which presented minimal contact angles, which were further reduced by the addition of plasticizers, increasing the hydrophilicity of the system (SDS7 and HSD7 in batches). The wettability investigation of the tablet blend revealed a greater reduction in the contact angle, which was attributable to the inclusion of additional hydrophilic matrix-forming excipients in the tablet formulation.

#### 3.4.8. In Vitro Drug Release Study

##### Effect of Drug Loading and Processing Method (SD and H on Drug Release)

**Figure 9:**
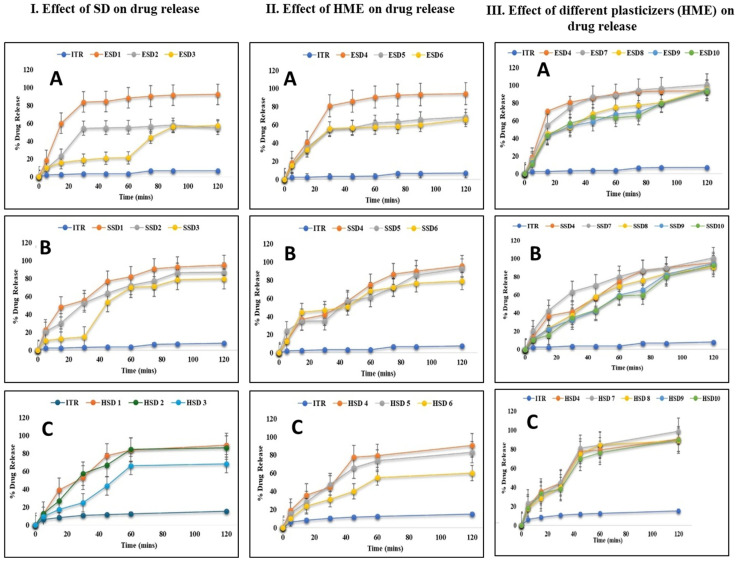
In vitro drug release of ITR ASDs. (**I**) Effect of SD drug release. (**II**) Effect of HME on drug release. (**III**) Effect of plasticizers on drug release (n = 3) ((**A**) = Eudragit^®^ EPO, (**B**) = Soluplus^®^, and (**C**) = AQOAT^®^ AS-HG).

The dissolution behavior of itraconazole (ITR) amorphous solid dispersions (ASDs) was evaluated with Eudragit^®^ EPO, Soluplus^®^, and AQOAT^®^ AS-HG across varying drug/polymer ratios and two manufacturing methods: spray drying (SD) and hot-melt extrusion (HME). As presented in Figure 9I,II and Table 4, increasing polymer content generally enhanced drug release, though the extent and mechanisms differed by polymer and process. For spray-dried EPO systems, increasing polymer content from a 2:1 to 1:2 ITR:EPO ratio enhanced release from 55.24 ± 0.2% to 92.28 ± 0.24% (Figure 9I(A)). The observed improvement can be attributed to increased polymer availability for drug stabilization and a reduction in recrystallization [80,81,82,83]. The high porosity and low density typical of spray-dried particles also contributed to faster initial dissolution by enabling rapid media penetration and drug diffusion. While in HME formulations, the 2:1 ITR:EPO ratio resulted in the greatest release (94.4 ± 0.09%), outperforming the 1:1 (68.98 ± 0.3%) and 1:2 (65.88 ± 0.06%) ITR:EPO combinations (Figure 9II(A)). This suggests that thermal and shear forces in HME enabled sufficient drug dispersion and stabilization, even at higher loading, possibly due to favorable ionic or hydrogen bonding facilitated by the low glass transition temperature of EPO.

While Piccinni et al. reported limited miscibility and recrystallization risks in ITZ/EPO solid dispersions at 30–40% loading, the present study demonstrated reduced crystallinity and >95% dissolution at pH 1.2 from ITZ/EPO (1:2) solid dispersions, with stable amorphous conversion, indicating enhanced solubilization and process performance [38].

Soluplus^®^-based ASDs exhibited consistent, ratio-dependent improvement in both methods. SD formulations increased from 79.5 ± 0.06% (2:1) to 94.86 ± 0.04% (1:2), while HME batches showed >93% release at 1:1 and 1:2 ratios. The amphiphilic nature of Soluplus^®^ supports dual-mode solubilization: hydrophobic interactions encapsulate ITR, while hydrophilic segments enhance medium uptake (Figure 9I(B)). HME further amplifies drug–polymer miscibility through melt mixing, resulting in near-complete release with both 1:1 (93.56 ± 0.02%) and 1:2 (96.81 ± 0.12%) ITR/polymer loading (Figure 9II(B)). However, at a 2:1 drug load, HME release dropped to 78.99 ± 0.04%, likely due to limited polymer capacity to maintain the drug in amorphous form, leading to phase separation or crystallinity (Figure 9II(B) [64,84]). While Davis et al. explored itraconazole dissolution enhancement using combinations of HPMC P and Soluplus^®^, the present study demonstrated >96% drug release within 2 h from ITZ/Soluplus^®^ hot-melt extrudates at both 1:1 and 1:2 ratios using Soluplus^®^ alone, highlighting its standalone capability for effective dissolution enhancement [85].

AQOAT^®^ AS-HG systems followed similar trends. In SD batches, dissolution improved from 68.43 ± 0.19% (2:1) to 89.22 ± 0.21% (1:2) (Figure 9I(C)), while HME batches showed 60.22 ± 0.12% to 93.70 ± 0.08% across the same ratios (Figure 9II(C)). The enteric nature of AQOAT^®^ AS-HG limited drug release in acidic pH and enables targeted intestinal delivery through pH-triggered swelling and erosion. Higher polymer ratios ensure more complete amorphization and pH-responsive matrix formation. HME further improves structural coherence and drug entrapment, enhancing release in neutral pH environments. Darwich et al. reported enhanced pH-dependent release using ITZ:HPMC AS-LF, while the present study demonstrated a more robust enteric-targeted performance, with ITZ:HPMC AS-HG (1:2) with higher drug loading, achieving <10% release in 0.1 N HCl and >85% controlled release in pH 6.8 within 2 h, indicating improved acid resistance and intestinal dissolution [86]. These results highlight that while increased polymer content universally benefits dissolution, the interaction between polymer chemistry and processing determines drug dispersion, stabilization, and release. HME generally provided superior performance, particularly at optimal drug/polymer ratios, due to enhanced molecular interactions and matrix integrity.

##### Effects of Different Plasticizers on Drug Release

To enhance processing and dissolution performance, plasticizers—propylene glycol (PG), triethyl citrate (TEC), Polysorbate 80, and Sorbitan monolaurate 20—were incorporated at 5–10% *w*/*w*. This range was chosen to lower the glass transition temperature (Tg) of the polymer matrix and increase polymer chain mobility during extrusion. A 5% concentration proved optimal, producing extrudates with sufficient plasticity for milling while maintaining mechanical integrity. Among the plasticizers tested, propylene glycol consistently demonstrated the most favorable impact on drug release, particularly in formulations containing Eudragit^®^ EPO, Soluplus^®^, and AQOAT^®^ AS-HG. In EPO-based spray-dried systems, PG and TEC showed similar performance, but in Soluplus^®^ and AQOAT^®^ AS-HG formulations, PG outperformed others (Figure 9III(A–C), Table 4). This enhancement is attributed to PG’s superior miscibility and its capacity to plasticize both hydrophilic and hydrophobic polymer segments, resulting in a more homogenous drug distribution. Mechanistically, PG facilitates improved release by reducing Tg, enhancing matrix flexibility, and increasing free volume, which collectively promote faster water penetration, polymer swelling, and drug diffusion. These effects are especially significant in polymers with amphiphilic or pH-responsive properties, where optimal plasticization directly translates to better dissolution performance.

##### Effect of Residence Time on Drug Release on Drug Amorphization and Release

To explore the impact of thermal exposure duration during hot-melt extrusion (HME), residence time, defined as the duration for which the formulation remains within the barrel, was varied (0, 5, and 10 min). Figure S3 represents the effect of residence time on drug release. While drug release showed consistent or slight improvement with increased residence time, a supplementary study to PXRD data analysis revealed a significant reduction in crystallinity from ~30% to ~21%, as shown in Table 5. This suggests that prolonged residence facilitates enhanced molecular mixing and more efficient drug amorphization due to sustained thermal and shear input. A 5 min residence time provided an optimal balance, ensuring improved amorphization while maintaining process efficiency. These findings underscore the importance of residence time as a critical yet often overlooked process parameter influencing the internal structure and performance of HME-based amorphous solid dispersions.

##### Drug Release of Optimized ASD-Loaded Tablets

**Figure 10:**
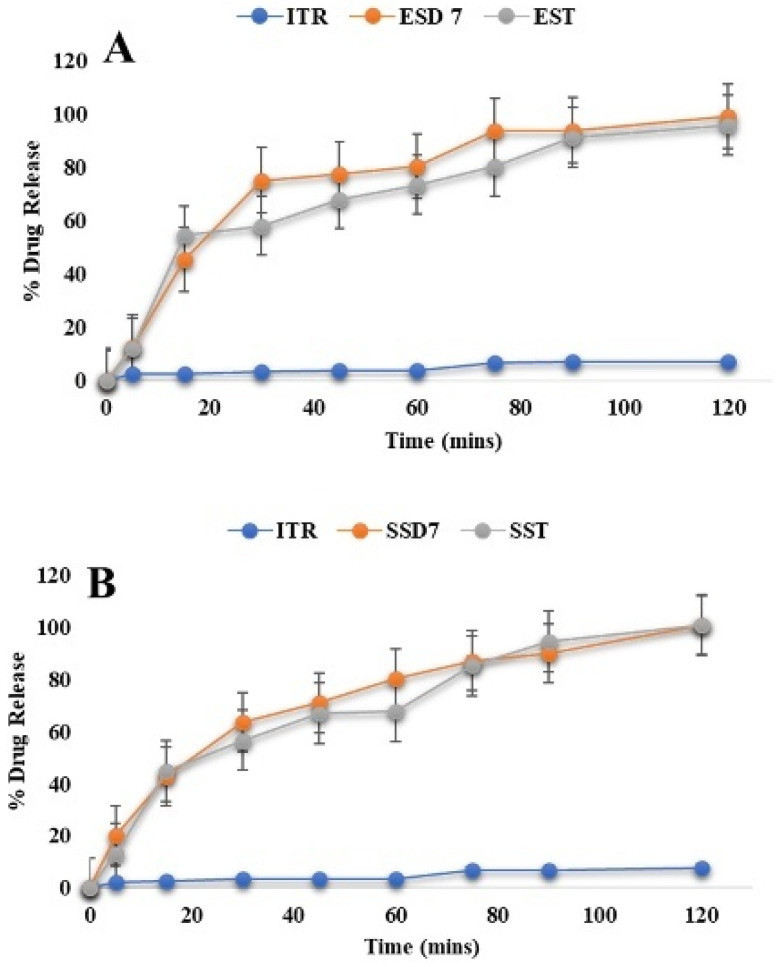

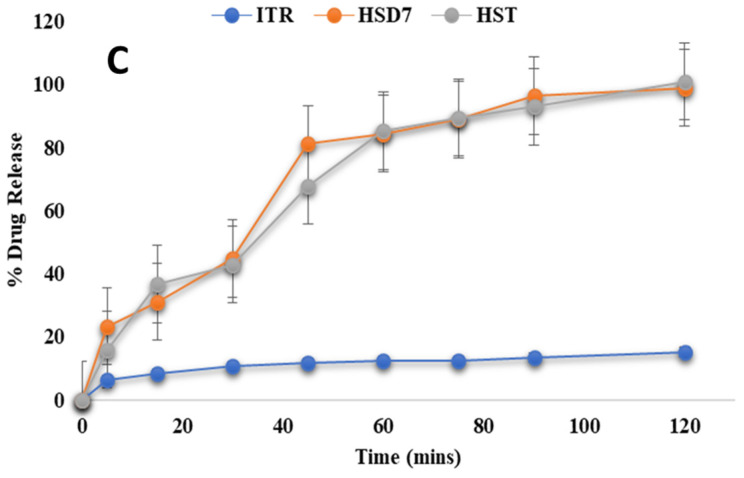
In vitro drug release of ASD-loaded tablets: ITR Eudragit^®^ EPO ASD Tablets (**A**), ITR Soluplus^®^ ASD Tablets (**B**), and ITR HPMC AS ASD Tablets (**C**).

The three different optimized ASD tablets (EST, SST, and HST) were evaluated, and results matched with pharmacopeia standards (data shown in Table 6). In vitro drug release of ASD-loaded tablets, namely ITR Eudragit^®^ EPO ASD Tablets (A), ITR Soluplus^®^ ASD Tablets (B), and ITR HPMC AS ASD Tablets (C), is shown in Figure 10. Release Kinetics for ITR ASD Tablets is explained in Table 7.

An optimized batch of ITR Eudragit^®^ EPO ASD-loaded tablets showed 95.88 ± 0.12% release within 2 h, following the Weibull model (R^2^ > 0.99), indicating an erosion-dominated release process consistent with surface-based matrix erosion facilitated by polymer hydration and disintegration [87,88,89] (Figure 10A). However, tablets containing ITR/Soluplus^®^ and ITR:AQOAT^®^ AS-HG ASDs demonstrated complete drug release (>100%) within 2 h, fitting best to the Peppas–Sahlin model (R^2^ > 0.99), indicative of a non-Fickian mechanism involving both diffusion and polymer matrix relaxation [90] (Figure 10B,C). The release kinetics for ITR ASD Tablets are shown in Table 6, and further details are shown in Supplementary Section S1. For Soluplus^®^, its amphiphilic character facilitates simultaneous water uptake and drug diffusion, while its flexible polymeric chains allow for relaxation-driven release. AQOAT^®^ AS-HG, being pH-sensitive, restricted release under gatric conditions (9.8 ± 0.8% at pH 1.2). The swelling and relaxation of the HPMC matrix at higher pH facilitated diffusion and contributed to sustained drug release [91]. These model fits provide strong mechanistic evidence that drug release is governed not only by polymer solubility and structure but also by polymer chain dynamics under physiological conditions. The alignment of empirical data with model predictions strengthens the rationale in the design strategy for pH-targeted or rapid-release formulations [92].

## 4. Conclusions and Future Prospects

In this study, we provide insights into manufacturing aspects and comprehensive characterization to gain a better understanding of ITR-loaded ASDs. The qualitative and quantitative selection of the solid dispersion carriers contributes crucially to the increase in the dissolution rate. The carriers helped maintain the drug in the amorphous state, enabling its utilization in the solid and solution states. The establishment of ASDs for all the drug carrier combinations was validated via XRPD, DSC, PLM, and SEM analyses. Among the ITR/polymer ratios of 1:2, 1:1, and 2:1, the 1:2 ratio resulted in an enhanced dissolution profile for all the extrudable polymers, including Eudragit^®^ EPO, Soluplus^®^, and AQOAT^®^ AS-HG. However, because of inadequate drug–polymer mixing and/or interaction, at higher API ratios, the drug exhibited a slow dissolution rate independent of polymer dissolution, resulting in inferior dissolution performance. The optimized ASD-loaded tablet formulations were successfully developed. In conclusion, our results reveal the advantages of HME-triggered amorphization as a continuous process for significantly improving solubility as compared to conventional techniques such as spray drying. However, animal studies, including pharmacokinetic and pharmacodynamic investigations, are desirable to provide a systematic framework for the future development of advanced oral drug delivery systems.

## Figures and Tables

**Table 1:** Hanson solubility parameter values.

Sr. No.	Component	Total Solubility Parameter: δ_T_	Total Solubility Parameter Difference: Δδ (MPa^½^)
1.	ITR	23.35	-
2.	Eudragit^®^ EPO	16.72	6.33
3.	Kollidon VA 64	20.56	2.79
4.	Soluplus^®^	19.35	4.00
5.	HPMC AS LG	26.18	2.83
6.	HPMC AS MG	26.15	2.80
7.	HPMC AS HG	26.11	2.76

**Table 2:** Experimental Design of the Film Casting Method.

Polymer	ITR/Polymer Ratio	Drying at Room Temperature (Primary Stage)	Redissolving Film and Drying at 50 °C (Secondary Stage)
Soluplus^®^	1:2	Transparent film	Transparent
	1:1	Transparent film	Transparent
	2:1	Transparent film	Turbid
Eudragit^®^ EPO	1:2	Transparent film	Transparent
	1:1	Turbid film	Turbid
	2:1	Turbid film	Turbid
Kollidon VA 64	1:2	Transparent film	Transparent
	1:1	Transparent film	Slightly turbid
	2:1	Turbid film	Turbid
HPMC AS LG	1:2	Transparent film	Transparent
	1:1	Turbid film	Turbid
	2:1	Turbid film	Turbid
HPMC AS MG	1:2	Transparent film	Transparent
	1:1	Transparent film	Slightly turbid
	2:1	Turbid film	Turbid
AQOAT^®^ AS-HG	1:2	Transparent film	Transparent
	1:1	Transparent film	Transparent
	2:1	Turbid film	Turbid

**Table 3:** Gas-Phase and Solution-Phase Energy Calculations.

Sr. No.	Combination	Gas Phase Energy (kcal/mol)	Solution Phase Energy (kcal/mol)	Binding Energy (kcal/mol)
1	Itraconazole	–3016.38	–3016.50	–
2	Soluplus^®^	–1451.95	–1451.97	–
3	ITR:Soluplus^®^ 1:1	–4263.75	–4263.88	204.59
4	ITR:Soluplus^®^ 1:2	–5919.82	–5919.90	0.54
5	ITR:Soluplus^®^ 2:1	–7483.81	–7483.93	1.04
6	Eudragit^®^ EPO	–979.75	–979.78	–
7	ITR:Eudragit^®^ EPO 1:1	–4289.00	–4289.10	–292.82
8	ITR:Eudragit^®^ EPO 1:2	–5753.76	–5753.76	–777.70
9	ITR:Eudragit^®^ EPO 2:1	–6482.18	–6482.46	+530.32
10	AQOAT^®^ AS-HG	–3441.31	–3441.44	–
11	ITR:AQOAT^®^ AS-HG 1:1	–6383.34	–6383.47	74.47
12	ITR:AQOAT^®^ AS-HG 1:2	–9981.21	–9981.43	–82.05
13	ITR:AQOAT^®^ AS-HG 2:1	–9550.45	–9550.63	–76.19

**Table 4:** In vitro drug release study of ITR ASDs (n = 3).

Batch	Drug/Polymer Ratio	Technique	Plasticizer	Cumulative Percent Release (%)
ESD1	ITR:EPO (1:2)	SD	-	92.28 ± 0.24
ESD2	ITR:EPO (1:1)	SD	-	57.02 ± 0.44
ESD3	ITR:EPO (2:1)	SD	-	55.24 ± 0.20
ESD4	ITR:EPO (1:2)	HME	-	94.4 ± 0.090
ESD5	ITR:EPO (1:1)	HME	-	68.98 ± 0.30
ESD6	ITR:EPO (2:1)	HME	-	65.88 ± 0.06
ESD7	ITR:EPO (1:2)	HME	PG	97.89 ± 0.10
ESD8	ITR:EPO (1:2)	HME	Triethyl citrate	95.88 ± 0.02
ESD9	ITR:EPO (1:2)	HME	Polysorbate 80	94.74 ± 0.67
ESD10	ITR:EPO (1:2)	HME	Sorbitan monolaurate 20	92.88 ± 0.05
SSD1	ITR:Soluplus^®^ (1:2)	SD	-	94.86 ± 0.04
SSD2	ITR:Soluplus^®^ (1:1)	SD	-	87.04 ± 0.08
SSD3	ITR:Soluplus^®^ (2:1)	SD	-	79.5 ± 0.06
SSD4	ITR:Soluplus^®^ (1:2)	HME	-	96.81 ± 0.12
SSD5	ITR:Soluplus^®^ (1:1)	HME	-	93.56 ± 0.02
SSD6	ITR:Soluplus^®^ (2:1)	HME	-	78.99 ± 0.04
SSD7	ITR:Soluplus^®^ (1:1)	HME	PG	97.11 ± 0.12
SSD8	ITR:Soluplus^®^ (1:1)	HME	Triethyl citrate	90.88 ± 0.02
SSD9	ITR:Soluplus^®^ (1:1)	HME	Polysorbate 80	94.74 ± 0.71
SSD10	ITR:Soluplus^®^ (1:1)	HME	Sorbitan monolaurate 20	92.88 ± 0.05
HSD1	ITR:AQOAT^®^ AS-HG (1:2)	SD	-	89.22 ± 0.21
HSD2	ITR:AQOAT^®^ AS-HG (1:1)	SD	-	83.50 ± 0.18
HSD3	ITR:AQOAT^®^ AS-HG (2:1)	SD	-	68.43 ± 0.19
HSD4	ITR:AQOAT^®^ AS-HG (1:2)	HME	-	93.70 ± 0.08
HSD5	ITR:AQOAT^®^ AS-HG (1:1)	HME	-	87.06 ± 0.16
HSD6	ITR:AQOAT^®^ AS-HG (2:1)	HME	-	60.22 ± 0.12
HSD7	ITR:AQOAT^®^ AS-HG (1:2)	HME	PG	94.44 ± 0.28
HSD8	ITR:AQOAT^®^ AS-HG (1:2)	HME	Triethyl citrate	88.82 ± 0.80
HSD9	ITR:AQOAT^®^ AS-HG (1:2)	HME	Polysorbate 80	90.42 ± 0.20
HSD10	ITR:AQOAT^®^ AS-HG (1:2)	HME	Sorbitan monolaurate 20	89.88 ± 0.03

**Table 5:** Effect of the residence time on the ITR ASD parameters (n = 3).

Batch	Residence Time	Drug Release (%)	Crystallinity (%)
ESD7	0 min	97.89 ± 0.10	30.1
	5 min	99.21 ± 0.11	24.9
	10 min	100.81 ± 0.22	22.5
SSD7	0 min	97.11 ± 0.06	30
	5 min	99.91 ± 0.71	22.6
	10 min	104.2 ± 0.02	21.5
HSD7	0 min	94.44 ± 0.09	25.7
	5 min	98.81 ± 0.28	24.9
	10 min	100.35 ± 0.8	23.6

**Table 6:** Evaluation Parameters for ITR ASD Tablets (Mean ± SD).

Batch	Weight (mg)	Hardness (kg/cm^2^)	Friability (%)	Disintegration (min)
EST	502.6 ± 1.8	5.4 ± 0.2	0.28% ± 0.01	11.2 ± 0.4
SST	498.9 ± 2.1	6.1 ± 0.3	0.35% ± 0.08	10.6 ± 0.5
HST	500.8 ± 1.5	5.7 ± 0.2	0.12% ± 0.02	12.0 ± 0.3

**Table 7:** Release Kinetics for ITR ASD Tablets.

Model	Rsqr_adj		
	EST (Eudragit^®^ EPO)	SST (Soluplus^®^)	HST (AQOAT^®^ AS-HG)
Zero-order	0.56	0.72	0.72
First-order	0.96	0.96	0.97
Higuchi	0.93	0.97	0.97
Korsmeyer–Peppas	0.93	0.97	0.96
Hixson–Crowell	0.98	0.94	0.98
Hopfenberg	0.93	0.93	0.97
Weibull	0.99	0.96	0.97
Peppas–Sahlin	0.97	0.99	0.99

## Data Availability

The data presented in this study are available through the whole manuscript and Supplementary Materials.

## Supplementary Materials

The following supporting information can be downloaded at https://www.mdpi.com/article/10.3390/pharmaceutics17091090/s1. Figure S1: Effect of drug loading on torque generation. Figure S2: Effect of temperature on torque generation. Figure S3: Effect of residence time on drug release. Supplementary Section S1. Release Kinetics for ITR ASD Tablets.
