# Supplementary material for: Continuous Processing Strategies for Amorphous Solid Dispersions of Itraconazole: Impact of Polymer Selection and Manufacturing Techniques
Source: Pharmaceutics. 2025 Aug 22;17(9):1090. doi: 10.3390/pharmaceutics17091090 (PMC12473836; doi:10.3390/pharmaceutics17091090)
Supplement: Supplementary file 1 [file pharmaceutics-17-01090-s001.zip › pharmaceutics-3784420-supplementary.pdf]

SUPPLEMENTARY MATERIAL

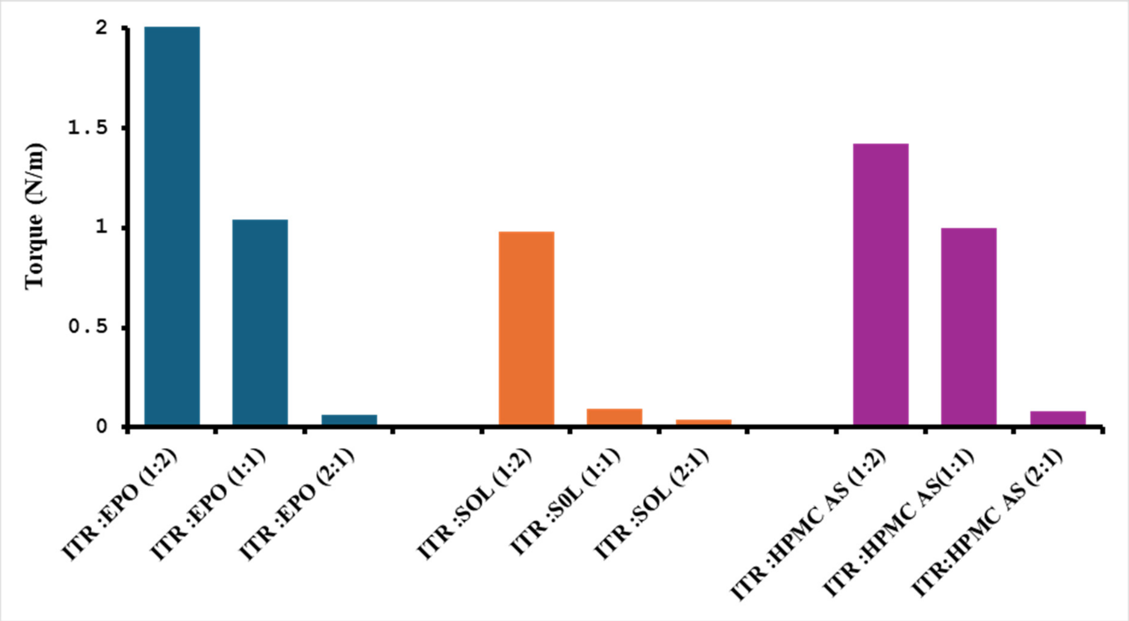

Figure S1. Effect of drug loading on torque generation

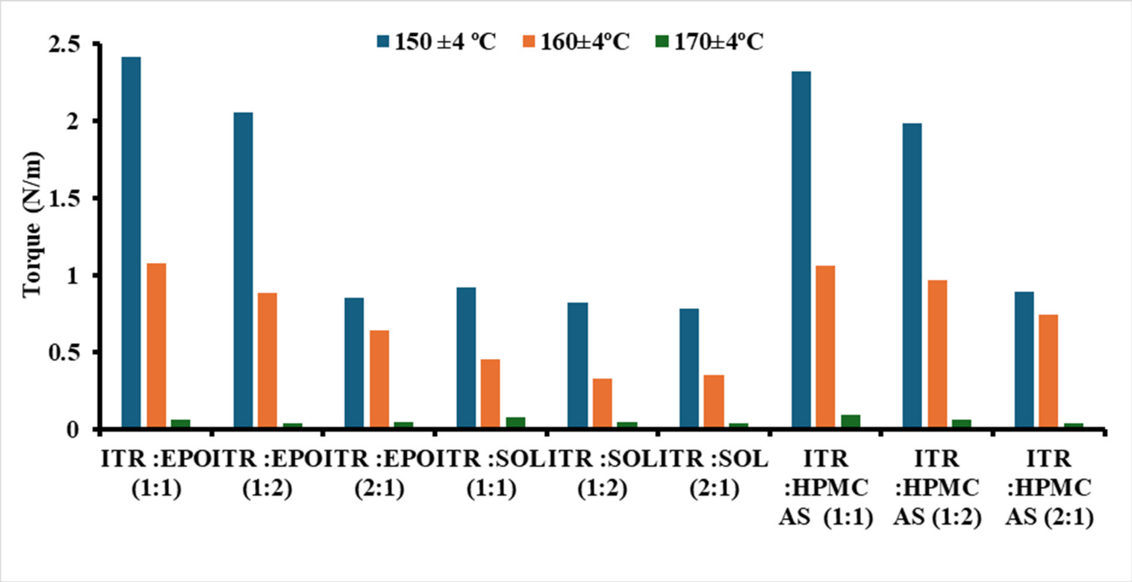

Figure S2. Effect of temperature on torque generation

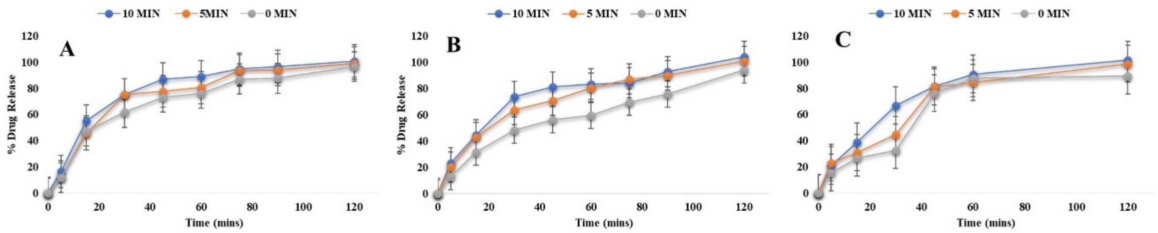

Figure S3. Effect of residence time on drug release

Supplementary Section S1. Release Kinetics for ITR ASD Tablets

1.ITR Eudragit EPO ASD loaded Tablet

***DD*Solver 1.0**	***Dissolution Data Modeling of Weibull Model***
Time Unit	min
Model	Weibull
Equation	F=100*{1-Exp[-((t-Ti)^β)/α]}

Best-fit Values

Parameter	No.1	Mean	SD	RSD(%)
α	132.094	132.094		
β	1.172	1.172		
Ti	0.267	0.267		

Secondary Parameter

Parameter	No.1	Mean	SD	RSD(%)
T25	22.521	22.521		
T50	47.381	47.381		
Td	64.670	64.670		
T75	85.361	85.361		
T80	96.913	96.913		

Goodness of Fit

Parameter	No.1
DF	6
R_obs-pre	0.9950
Rsqr	0.9900
Rsqr_adj	0.9867
MSE	14.0051
MSE_root	3.7423
Weighting	1
SS	84.0304
WSS	84.0304
AIC	45.8806
MSC	3.6794

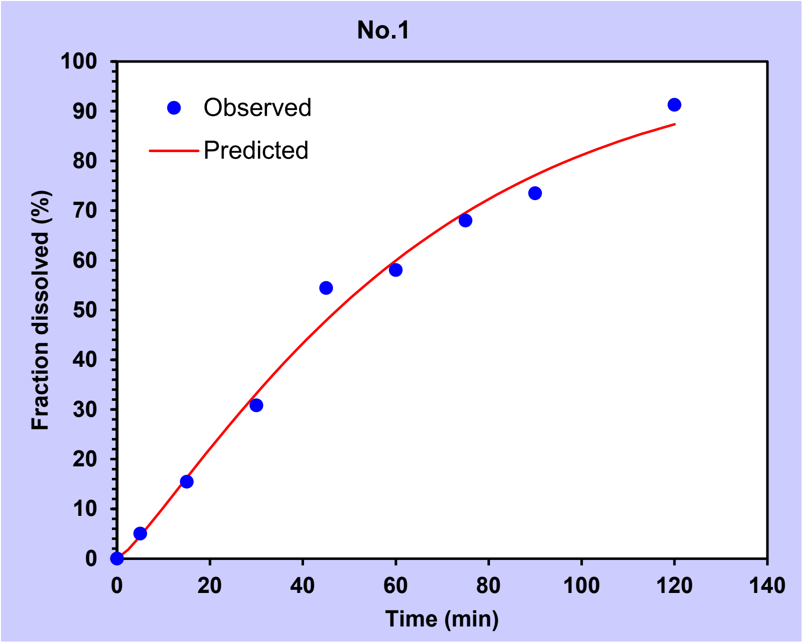
No.1
Observed
Predicted
Fraction dissolved (%)
Time (min)
0
10
20
30
40
50
60
70
80
90
100
0
20
40
60
80
100
120
140

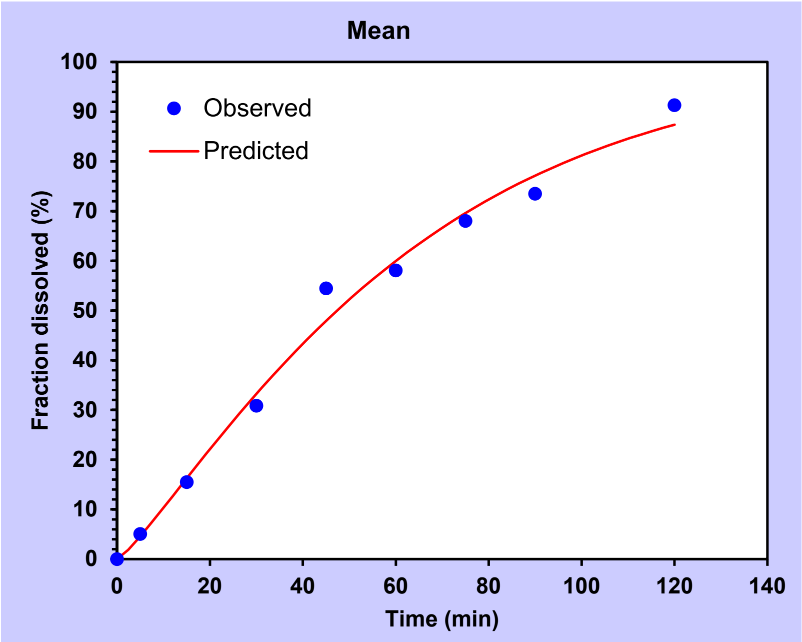
Mean
Observed
Predicted
Fraction dissolved (%)
Time (min)
0
10
20
30
40
50
60
70
80
90
100
0
20
40
60
80
100
120
140

2. ITR Soluplus ASD loaded Tablet

***DD*Solver 1.0**	***Dissolution Data Modeling of Peppas-Sahlin Model***
Time Unit	min
Model	Peppas-Sahlin
Equation	F=k1*t^m+k2*t^(2*m)

Best-fit Values

Parameter	No.1	Mean	SD	RSD(%)
k1	9.030	9.030		
k2	-0.213	-0.213		
m	0.635	0.635		

Secondary Parameter

Parameter	No.1	Mean	SD	RSD(%)
T25	5.570	5.570		
T50	19.257	19.257		
T75	45.700	45.700		
T80	54.039	54.039		
T90	78.848	78.848		

Goodness of Fit

Parameter	No.1
DF	6
R_obs-pre	0.9958
Rsqr	0.9916
Rsqr_adj	0.9887
MSE	12.7928
MSE_root	3.5767
Weighting	1
SS	76.7570
WSS	76.7570
AIC	45.0658
MSC	3.5413

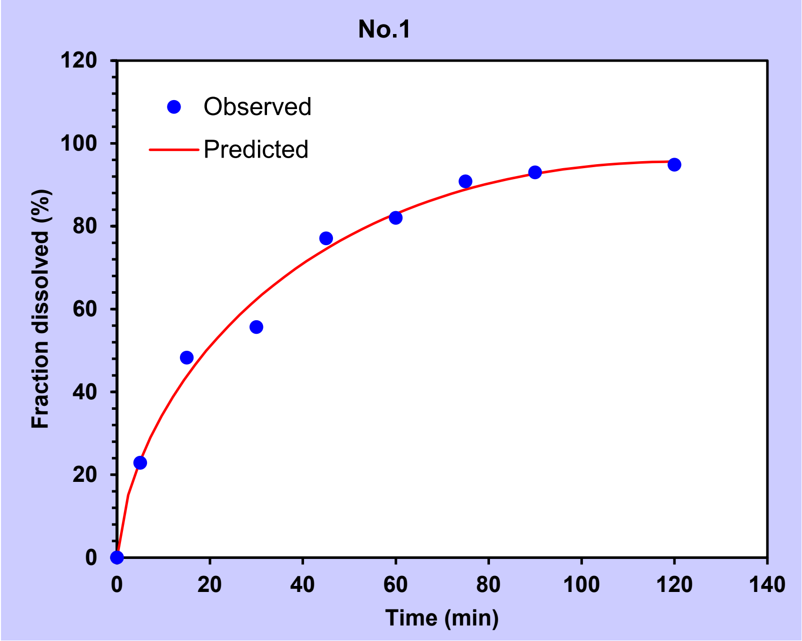

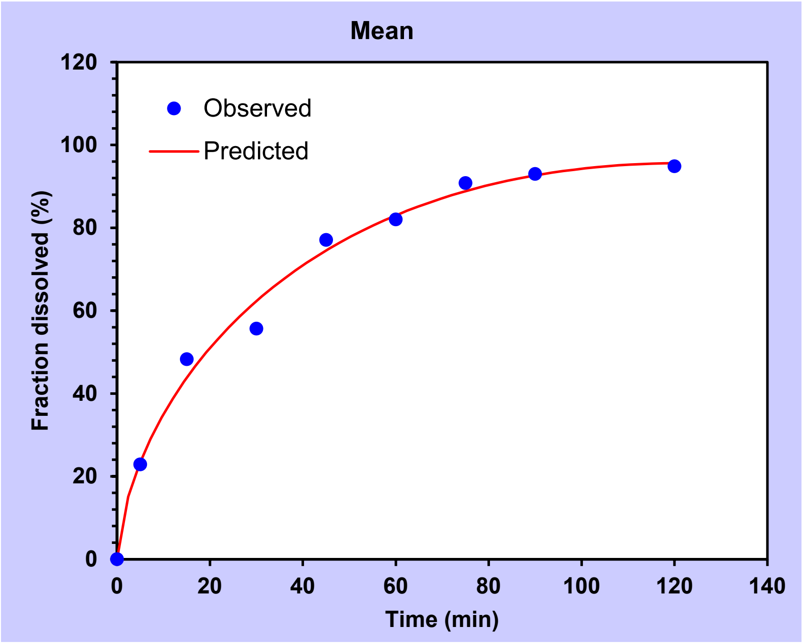
Mean
Observed
Predicted
Fraction dissolved (%)
Time (min)
0
20
40
60
80
100
120
140

3. ITR HPMC AS ASD loaded Tablet

DDSolver 1.0	*Dissolution Data Modeling of Peppas-Sahlin Model*
Time Unit	min
Model	Peppas-Sahlin
Equation	F=k1*t^m+k2*t^(2*m)

Best-fit Values

Parameter	No.1	Mean	SD	RSD(%)
k1	2.371	2.371		
k2	-0.014	-0.014		
m	0.970	0.970		

Secondary Parameter

Parameter	No.1	Mean	SD	RSD(%)
T25	12.179	12.179		
T50	27.228	27.228		
T75	47.039	47.039		
T80	52.069	52.069		
T90	64.366	64.366		

Goodness of Fit

Parameter	No.1
DF	6
R_obs-pre	0.9990
Rsqr	0.9978
Rsqr_adj	0.9971
MSE	4.4959
MSE_root	2.1204
Weighting	1
SS	26.9754
WSS	26.9754
AIC	35.6543
MSC	5.1179

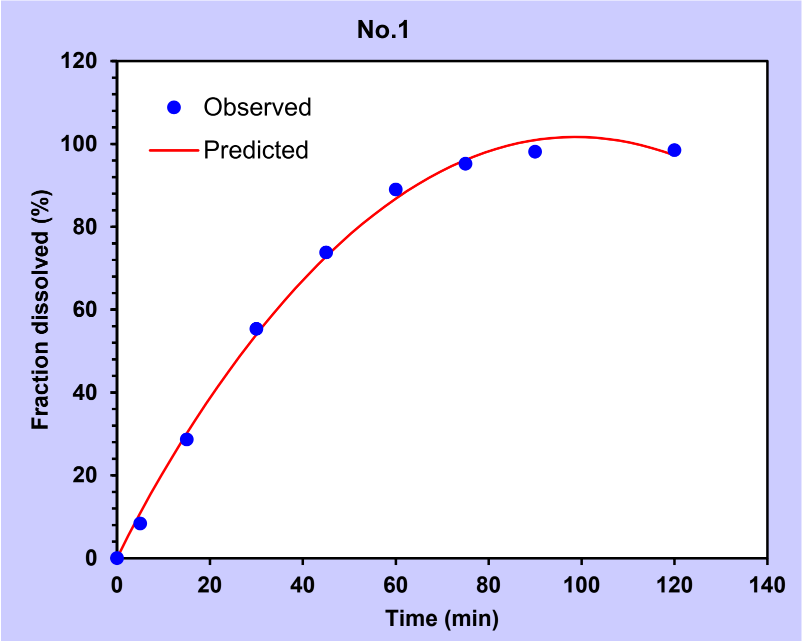

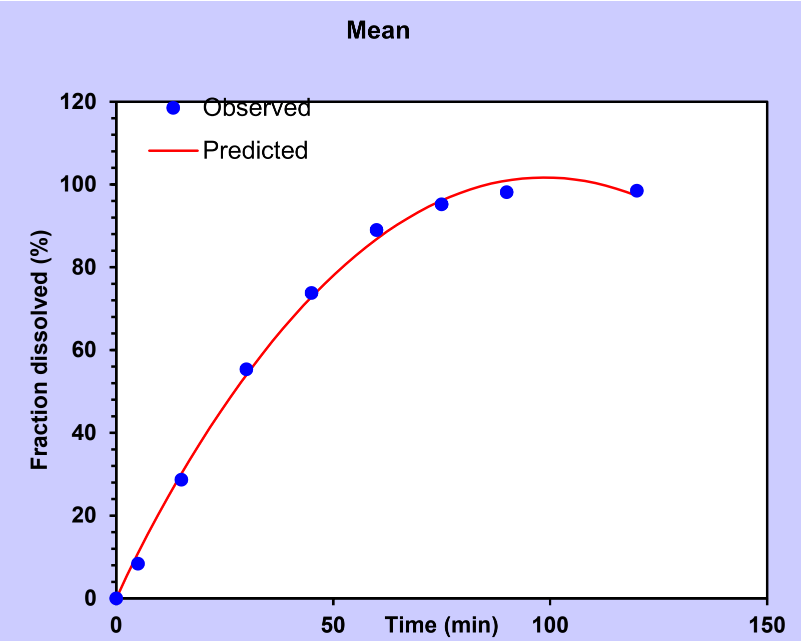
Mean
Observed
Predicted
Fraction dissolved (%)
120
100
80
60
40
20
0
0
50
Time (min)
100
150
